# Mitochondrial Dysfunction Is Inducible in Lymphoblastoid Cell Lines From Children With Autism and May Involve the TORC1 Pathway

**DOI:** 10.3389/fpsyt.2019.00269

**Published:** 2019-05-07

**Authors:** Sirish C. Bennuri, Shannon Rose, Richard E. Frye

**Affiliations:** ^1^Arkansas Children’s Research Institute and Department of Pediatrics, University of Arkansas for Medical Sciences, Little Rock, AR, United States; ^2^Barrow Neurologic Institute at Phoenix Children’s Hospital, Phoenix, AZ, United States; ^3^Department of Child Health, University of Arizona College of Medicine—Phoenix, Phoenix, AZ, United States

**Keywords:** autism, mitochondria, mitoplasticity, mechanistic target of rapamycin, reactive oxygen species

## Abstract

We previously developed a lymphoblastoid cell line (LCL) model of mitochondrial dysfunction in autism spectrum disorder (ASD); some individuals with ASD showed mitochondrial dysfunction (AD-A) while other individuals (AD-N) demonstrated mitochondrial respiration similar to controls (CNT). To test the hypothesis that mitochondrial dysfunction could be a consequence of environmental exposures through chronic elevations in reactive oxygen species (ROS), we exposed LCLs to prolonged ROS. We also examined expression of metabolic regulatory genes and the modulating effect of the mechanistic target of rapamycin (mTOR) pathway. Prolonged ROS exposure induced or worsened mitochondrial dysfunction in all LCL groups. Expression of genes associated with ROS protection was elevated in both AD-N and AD-A LCLs, but mitochondrial fission/fusion and mitoplasticity gene expression was only increased in AD-N LCLs. Partial least squares discriminant analysis showed that mTOR, UCP2 (uncoupling protein 2), SIRT1 (sirtuin 1), and MFN2 (mitofusin-2) gene expression differentiated LCL groups. Low-dose rapamycin (0.1 nM) normalized respiration with the magnitude of this normalization greater for AD-A LCLs, suggesting that the mammalian target of rapamycin complex 1 (mTORC1) pathway may have a different dynamic range for regulating mitochondrial activity in individuals with ASD with and without mitochondrial dysfunction, potentially related to S6K1 (S6 kinase beta-1) regulation. Understanding pathways that underlie mitochondrial dysfunction in ASD may lead to novel treatments.

## Introduction

Mitochondria are involved in many essential cellular functions; besides production of adenosine triphosphate (ATP), mitochondria are essential for calcium buffering, redox regulation, apoptosis, and inflammation. Classic mitochondrial diseases are rare, but novel forms of mitochondrial dysfunction are believed to be associated with more common diseases, particularly those where the environment contributes to the etiology. Mitochondrial dysfunction is documented in psychiatric diseases (1–4), neurodegenerative disorders (5), persistent systemic inflammation (6), cardiac disease (7), cancer (8), and diabetes (9). Mitochondrial dysfunction is also closely associated with neurodevelopmental disorders, particularly autism spectrum disorder (ASD) (10, 11) and genetic syndromes closely associated with ASD including mechanistic target of rapamycin (mTOR) (12–15); phosphatase and tensin homolog (PTEN) (16) and WDR45 (17) mutations; Rett (18–20), Phelan–McDermid (21), Angelman (22), and Down (23, 24) syndromes; as well as 15q11-q13 duplication (25, 26) and septo-optic dysplasia (27).

Mitochondrial dysfunction in ASD is unique. First, the great majority (∼75%) of those with both ASD and mitochondrial disease *do not* show mitochondrial or nuclear genetic abnormalities, suggesting that the changes in mitochondrial function are either secondary to alterations in nonmitochondrial metabolic or regulatory pathways and/or due to changes in nonmitochondrial genes or epigenetic changes (11). Second, unlike classic mitochondrial disease, where mitochondrial activity is depressed, electron transport chain (ETC) activity in individuals with ASD has been reported to be elevated significantly above normal in muscle (28, 29), skin (30), gut mucosa (31), buccal epithelium (32–34), and brain (35).

In a series of studies, the authors have described and validated a lymphoblastoid cell line (LCL) model of mitochondrial dysfunction in samples obtained from individuals with ASD (36–42). In this model, about one-third of individuals with ASD (called AD-A) have LCLs with respiratory rates twice that of LCLs from control (CNT) individuals, while the remainder of the individuals with ASD (called AD-N) have LCLs with respiratory rates equivalent to LCLs from CNT individuals. Using the Mitochondrial Oxidative Stress Test (MOST) (43), we systematically increased reactive oxygen species (ROS) *in vitro* to demonstrate that AD-A LCLs are sensitive to physiological stress as they consistently show a depletion in reserve capacity (RC) as ROS is increased (36–42). In addition, we have demonstrated that mitochondrial respiration of AD-A LCLs responds differently to environmental toxicants (38, 40) and enteric short-chain fatty acids (39, 42) as compared to mitochondrial function in AD-N and CNT LCLs. In addition, we have linked this atypical mitochondrial function seen in AD-A LCLs to ASD behaviors; indeed, higher respiratory rates in the ASD LCLs were found to be associated with more severe repetitive behaviors measured on the gold-standard diagnostic tool for ASD, the Autism Diagnostic Observation Schedule (ADOS) (41).

The reason for the abnormal mitochondrial function in the AD-A LCLs is not obvious; we previously hypothesized that this elevation in mitochondrial respiration was an adaptive response that developed as a consequence of previous exposure(s) to chronic extrinsic and/or intrinsic stressors (37), a hypothesis that this paper is designed to test. Since many environmental stressors may have their biological effect through increases in ROS (21, 44–46) and since ROS is elevated in many ASD-derived tissues (45), including LCLs (37) and brain (47, 48), we hypothesized that prolonged environmental exposures, potentially through prolonged exposure to ROS, would alter long-term mitochondrial function by increasing respiratory rates. Since this hypothesis has never been tested directly, we will test this hypothesis in this paper by exposing ASD and CNT LCLs to prolonged ROS.

Thus, in this study, we determine whether prolonged exposure to ROS will increase mitochondrial respiratory rates in our LCL model so that LCLs with normal mitochondrial respiration (i.e., AD-N, CNT) will demonstrate increases in mitochondrial respiration and LCLs with increased respiration (i.e., AD-A) will further demonstrate increases in mitochondrial respiration. Showing that such a change can be induced would support the hypothesis that this change in mitochondrial respiration is inducible.

We also examined changes in molecular pathways associated with mitochondrial dysfunction by measuring the expression of genes, including those involved in regulating redox metabolism (uncoupling protein 2, UCP2; mitochondrial superoxide dismutase 2, SOD2) (49–51), mitochondrial response to stress (sirtuin 1, SIRT1; sirtuin 3, SIRT3; hypoxia-inducible factor 1-alpha, HIF1α; peroxisome proliferator-activated receptor gamma coactivator 1-alpha, PGC1α) (52–54), mitophagy (PTEN-induced putative kinase 1; PINK1), mitochondrial fusion (mitofusin-2, MFN2), mitochondrial fission (dynamin-1-like protein, DRP1), and those that can modulate cellular metabolism (AMP-activated protein kinase, AMPK; RAC-alpha serine/threonine-protein kinase, AKT1) and are associated with ASD (PTEN; mTOR) (55). We examined both the average expression across groups and the relationship between expressions of genes in various pathways.

Lastly, we examined the relationship of bioenergetic abnormalities found in ASD LCLs to the mTOR pathway for several reasons. First, abnormalities in mTOR complex 1 (mTORC1) signaling occurs in approximately 25% of individuals with nonsyndromic autism (56), which is a similar proportion of ASD LCLs with atypical bioenergetics (36, 37) (i.e., AD-A) in our model. Second, mTOR can influence mitochondrial function through several molecular pathways: mTORC1 inhibits eukaryotic translation initiation factor 4E-binding proteins (4EBP) that in turn releases inhibition of eIF4E and promotes translation of nuclear-encoded mitochondria-related messenger ribonucleic acids (mRNAs) and an increase in ETC activity (57) and mTORC1 positively influences ribosomal protein S6 kinase beta-1 (S6K1), which negatively regulates mitochondrial function through inhibition of the expression of such genes as UCP2, PGC1α, and DRP1 (58). Activation of S6K1 by mTORC1 is inhibited by low-dose rapamycin while high-dose rapamycin inhibits both S6K1 and 4EBP (59). Thus, as an additional experiment, we examined the effect of mTORC1 by determining whether low-dose rapamycin modulates mitochondrial respiration and whether such changes are more significant in the LCLs with higher respiratory rates (i.e., AD-A LCLs).

## Methods

### Lymphoblastoid Cell Lines and Culture Conditions

LCLs derived from white males diagnosed with autistic disorder (AD) were chosen from pedigrees with at least one other affected male sibling (i.e., multiplex family) [mean (SD) age, 8.5 (3.0) years]. These LCLs were obtained from the Autism Genetic Resource Exchange (Los Angeles, CA, USA) and the National Institute of Mental Health (Bethesda, MD, USA) center for collaborative genomic studies on mental disorders. All relevant guidelines and regulations were followed. These deidentified human samples were determined to be exempt from institutional review board (IRB) review by the University of Arkansas for Medical Science IRB. Donors were diagnosed using the ADOS or the Autism Diagnostic Interview–Revised (ADI-R), both gold-standard instruments.

In our previous studies (36–42), these LCLs were categorized into two different types: ones with atypical mitochondrial respiration (AD-A) and those with typical respiration (AD-N). These metabolic groupings are repeatable across several studies (36–42). All respiratory parameters were reviewed to ensure that the Seahorse runs for each LCL were consistent with their assigned group. Only one case was found where the Seahorse respiratory measurements of an LCL was not consistent with the expected group. One AD-N LCL (02C10618) demonstrated baseline respiratory parameters more consistent with AD-A LCLs in the rapamycin experiment, so it was excluded from the analysis.

CNT LCLs were derived from healthy white boys with no documented behavioral or neurological disorder and without any first-degree relative suffering from a medical disorder that might involve mitochondrial dysfunction [mean (SD) age, 8.5 (2.8) years]. CNT LCLs came from Coriell Cell Repository (Camden, NJ, USA).

The average cell passage was 12, with a maximum passage of 15, since genomic stability is very high at this low passage number. Cells were maintained in Roswell Park Memorial Institute (RPMI) 1640 culture medium with 15% fetal bovine serum and 1% penicillin/streptomycin (Invitrogen, Grand Island, NY, USA) in a humidified incubator at 37°C with 5% CO_2_.

### Seahorse Assay

**Figure 1:**
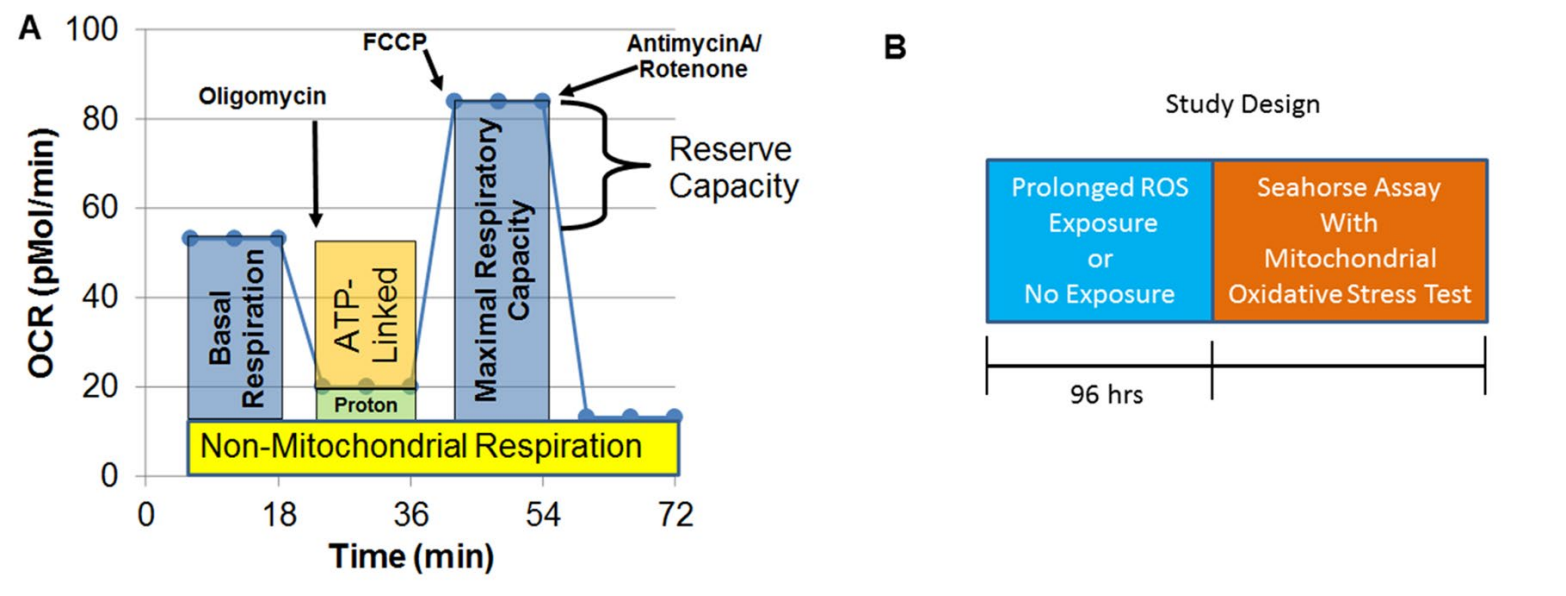
Seahorse assay and outline of the prolonged reactive oxygen species (ROS) exposure experiment. **(A)** Mitochondrial respiration is inferred by measuring oxygen consumption rate (OCR). OCR is measured three times over 18 min at each measurement phase. Initially, basal respiration is measured. The complex V inhibitor oligomycin is then used to determine how much of basal respiration is *ATP-linked respiration* and *proton leak respiration*. Then, a protonophore, carbonyl cyanide-*p*-trifluoromethoxyphenyl-hydrazon (FCCP) is used to drive the respiratory chain to its maximal rate by collapsing the inner membrane gradient, allowing *maximal respiratory capacity* to be determined. To determine non-mitochondrial respiration, antimycin A and rotenone, Complex III and I inhibitors, are added to stop the respiratory chain. *Reserve capacity* is calculated as the difference between basal respiration and maximal respiratory capacity. **(B)** Prolonged exposure (96 h) to a low concentration of DMNQ is used to simulate chronic exposure to a low level of ROS while other cells had no exposure to DMNQ as a control.

A Seahorse Extracellular Flux (XF) 96 Analyzer (Agilent Technologies, Santa Clara, CA, USA) measured oxygen consumption rate (OCR), an indicator of mitochondrial respiration, in live intact LCLs in real time. Each run (each line of **Supplementary Tables 1** and **2**) examined matched LCL groups (CNT, AD-N, AD-A) on the same plate to control for experimental variation in mitochondrial activity measurement. The assay (**Figure 1A**), which has been described previously (36–42), provides measures of *ATP-linked respiration (ALR), proton leak respiration (PLR), maximal respiratory capacity (MRC), and reserve capacity (RC)*.

### Mitochondrial Oxidative Stress Test

To determine the vulnerability of the mitochondria to acute ROS exposure, we developed the Mitochondrial Oxidative Stress Test (MOST) in our previous studies (43). We examined the sensitivity of LCLs to various increasing levels of ROS by systematically exposing LCLs to increasing concentrations of 2,3-dimethoxy-1,4-napthoquinone (DMNQ; Sigma-Aldrich, St. Louis, MO, USA) for 1 h prior to the Seahorse assay. LCLs were exposed to 0 μM (control no exposure), 5 μM, 10 μM, and 15 μM DMNQ since these concentrations of DMNQ provide a full range of ROS challenge such that the highest concentration (15 μM DMNQ) significantly reduced RC to its minimum in almost every LCL type. A 5 mg/mL DMNQ solution was diluted in DMEM XF assay media into a 10X stock and added to cells in an XF-PS plate in a non-CO_2_ incubator at 37°C 1 h prior to the Seahorse assay.

### Prolonged Low-Concentration Reactive Oxygen Species Exposure

To simulate prolonged exposure to low concentrations of ROS, 13 sets of LCLs (**Supplementary Table 1**) were cultured in 1 μM DMNQ for 96 h prior to the Seahorse assay or left untreated (0 μM) (**Figure 1B**). Thirteen pairs of AD-N and AD-A LCLs were matched with an age-matched male CNT LCL. Due to the low availability of CNT LCLs that fit our criteria, a single CNT LCL was matched with two ASD LCLs in two cases. Also, three AD-A LCLs were matched twice with AD-N LCLs. Matching was done to control for variations in measurement of mitochondrial function.

### Rapamycin Exposure

To determine the influence of the mTOR pathway, we first used both low (0.01 nM and 0.1 nM) and high (1 nM and 10 nM) concentrations of rapamycin with both 1 h and 2 h exposure times on one set of AD-A and AD-N LCLs to determine if there was a differential effect of low-dose rapamycin between the two cell lines and, if so, the optimal rapamycin concentration and exposure time. As a result of this titration, eight pairs of AD-A and AD-N LCLs (**Supplementary Table 2**) were exposed to 0.1 nM of rapamycin for 2 h prior to the Seahorse assay or left untreated (0 nM). Rapamycin was used as it preferentially inhibits the effect of mTORC1 (60), particularly on S6K1 at low dose (59).

### Gene Expression

Total RNA was isolated from 2 million CNT, AD-N, and AD-A LCLs (**Supplementary Table 3**) using the RNeasy mini kit (Qiagen, Hilden, Germany) following the manufacturer’s protocol. Complementary deoxyribonucleic acid (cDNA) synthesis (2 µg per 20-µL reaction mix) was performed using the High-Capacity cDNA Reverse Transcription Kit (Applied Biosystems, Waltham, MA, USA) as indicated by the manufacturer. Primers were designed using the online real-time polymerase chain reaction (PCR) tool from IDT DNA (www.idtdna.com/scitools/Applications/RealTimePCR/). **Supplementary Table S4** outlines the primer sequences. Quantitative PCR reactions were performed for all target genes using the Power SYBR Green PCR Master Mix (Applied Biosystems, Waltham, MA, USA) on an ABI 7900HT Fast Real Time PCR system. Relative quantification was performed to the housekeeping gene, HPRT1 (hypoxanthine phosphoribosyltransferase 1).

### Analytic Approach

To analyze group effects, a mixed-model regression was conducted *via* SAS version 9.3 (Cary, NC, USA) “glmmix” procedure. The mixed model allows matched samples on each Seahorse plate to be compared to one another while controlling for any variation associated with the Seahorse assay itself. The mitochondrial parameters were response variables with a between-group effect of LCL group (e.g., AD-N v AD-A v CNT) and within-group repeated factors of prolonged ROS exposure (exposed vs. nonexposed) and DMNQ concentration as well as the interaction between these effects. DMNQ concentration was a continuous variable. For all models, random effects included the intercept. *F* tests evaluated significance. Planned *post hoc* orthogonal contrasts, which are *t*-distributed, examined significant group effects. Data were normally distributed and variation was similar across groups. Graphs show standard error bars. Gene expression was similarly analyzed, although without the DMNQ concentration variable. Rapamycin exposure data were similarly analyzed, although the exposure variable was with rapamycin. We also performed partial least squares discriminant analysis (PLSDA) using R version 3.5.0 mixOmics Omics Data Integration Project version 6.3.2.

## Results

### Prolonged Exposure to Reactive Oxygen Species: Mitochondrial Function

To determine if prolonged exposure to low concentrations of ROS can alter mitochondrial function, potentially producing the atypical patterns of respiration seen in the AD-A LCLs, we exposed the three types of LCLs (CNT, AD-N, and AD-A) to a low concentration of DMNQ for 96 h or cultured normally (control). We then conducted the Seahorse assay including the MOST. This allowed us to determine if prolonged ROS exposure changed overall average respiration and/or the susceptibility of the mitochondria to acute ROS increases. Thus, we report the change in overall average respiration and the change in respiration with ROS challenge (as part of the MOST) as a result of prolonged (96 h) exposure to low levels of ROS.

#### Adenosine Triphosphate–Linked Respiration

**Figure 2:**
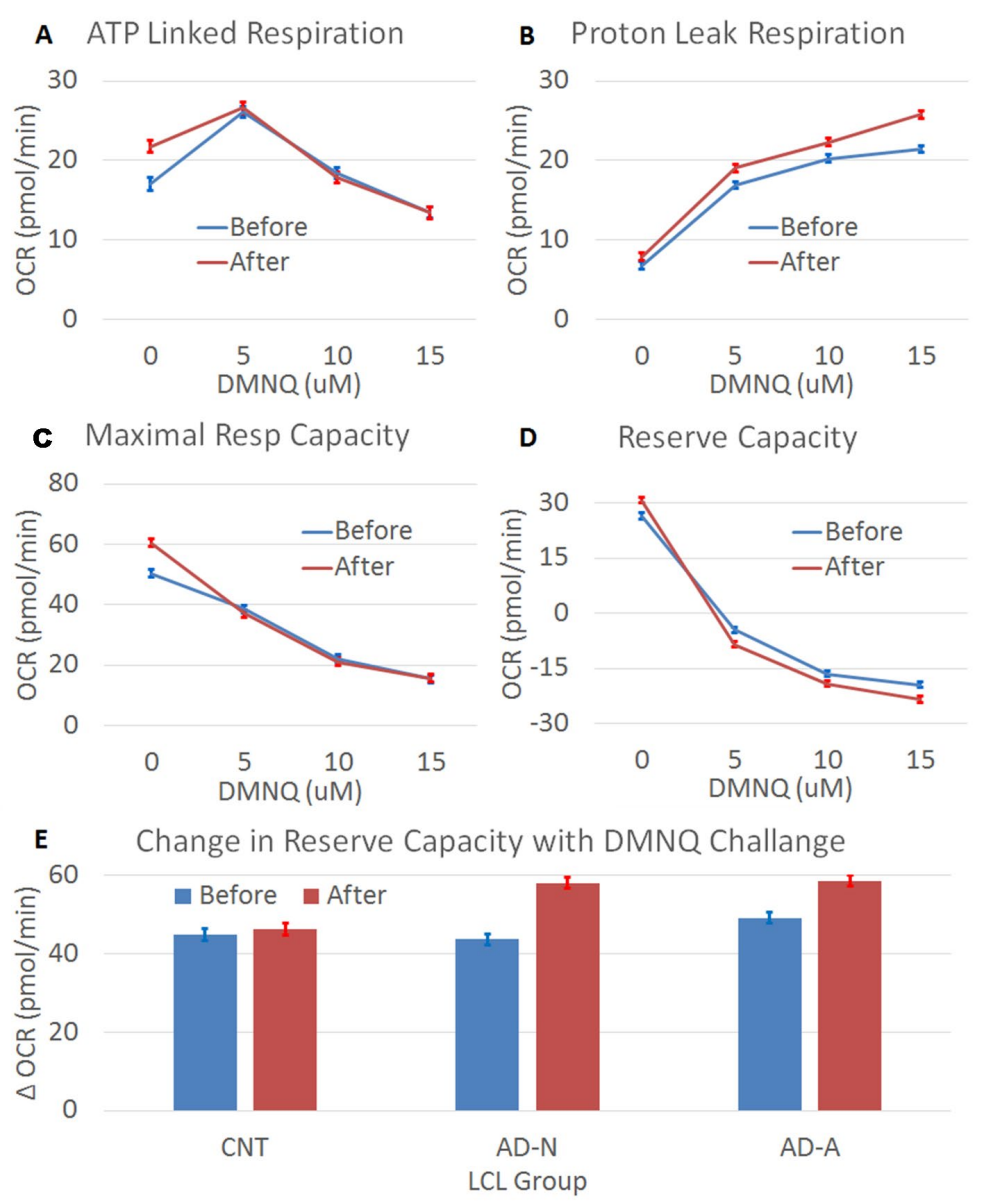
The effect of prolonged (96 h) ROS exposure on mitochondrial function in lymphobastoid cell lines (LCLs). In these expierments, 2,3-dimethoxy-1,4-napthoquinone (DMNQ) was used to increase ROS *in vitro* both during the prolonged incubation and 1 h prior to the mitochondrial measurements in order to determine the effect of acute increases in ROS on the LCLs. Prolonged ROS exposure (red) shifted mitochondrial function toward an atypical pattern that has been reported in a subset of LCLs derived from children with autistic disorder (AD). This includes increasing **(A)** ATP-linked respiration, **(B)** proton leak respiration, **(C)** maximal respiratory capacity, and **(D)** reserve capacity, along with a greater drop in reserve capacity when challanged with an acute increase in ROS. **(E)** The change in reserve capacity with increasing acute DMNQ concetration was different across the LCL groups, so the difference between the lowest (0 µM) and highest (15 µM) DMNQ concentrations is depicted in the bottom graph to demonstrate the interaction.

Prolonged ROS exposure increased overall ALR [*F*(1,1144) = 4.71, *p* < 0.05], with this effect not significantly different across LCL groups. The change in ALR with increasing DMNQ was changed by prolonged ROS exposure [*F*(1,1144) = 5.07, *p* < 0.01] without this change being significantly different across LCL groups (**Figure 2A**). Thus, prolonged ROS exposure changed mitochondrial respiration to have a higher baseline ALR and a greater change in ALR. This suggests that indeed prolonged ROS exposure shifts ALR toward the mitochondrial dysfunction abnormalities typically seen in the AD-A LCLs at baseline. Interestingly, these changes seem to be similar across LCL types, suggesting that it is a general adaptation of the mitochondria.

#### Proton Leak Respiration

Prolonged ROS exposure did influence overall PLR [*F*(1,1144) = 52.02, *p* < 0.001], but this change was not significantly different across LCL groups. The typical increase in PLR that occurs with redox challenge was amplified after prolonged ROS exposure [*F*(1,1144) = 3.95, *p* < 0.01] but this change was not significantly different across LCL groups (**Figure 2B**). Thus, prolonged ROS exposure changed mitochondrial respiration to have a greater change in PLR. This suggests that indeed prolonged ROS exposure shifts PLR toward the mitochondrial abnormalities seen in the AD-A LCLs at baseline.

#### Maximal Respiratory Capacity

Prolonged ROS exposure increased overall MRC [*F*(1,1144) = 4.97, *p* < 0.05], with this change not significantly different across LCL groups. The change (decrease) in MRC was accentuated after prolonged ROS exposure [*F*(1,1144) = 9.37, *p* < 0.0001], with this effect not significantly different across LCL groups (**Figure 2C**). Thus, prolonged ROS exposure changed mitochondrial respiration to increase overall MRC and cause a greater change in MRC. This suggests that indeed prolonged ROS exposure shifts MRC toward abnormalities seen in the AD-A LCLs at baseline.

#### Reserve Capacity

Prolonged ROS exposure increased overall RC [*F*(1,1144) = 7.81, *p* < 0.01], with this effect not significantly different across LCL groups. Prolonged ROS exposure also augmented the change (decrease) in RC [*F*(1,1144) = 11.55, *p* < 0.0001] (**Figure 2D**), with this effect significantly different across LCL groups [*F*(6,1144) = 2.37, *p* < 0.05] (**Figure 2E**). As can be seen in **Figure 2E**, the change in RC with the acute ROS challenge is greater for the AD groups than the CNT group. Thus, prolonged ROS exposure changed mitochondrial respiration to have a higher overall RC and greater change in RC, with some of these effects more prominent in the ASD LCLs. This suggests that indeed prolonged ROS exposure shifts RC toward abnormalities seen in the AD-A LCLs at baseline.

### Gene Expression Differences Across Lymphoblastoid Cell Line Groups: Statistical Differences

**Figure 3:**
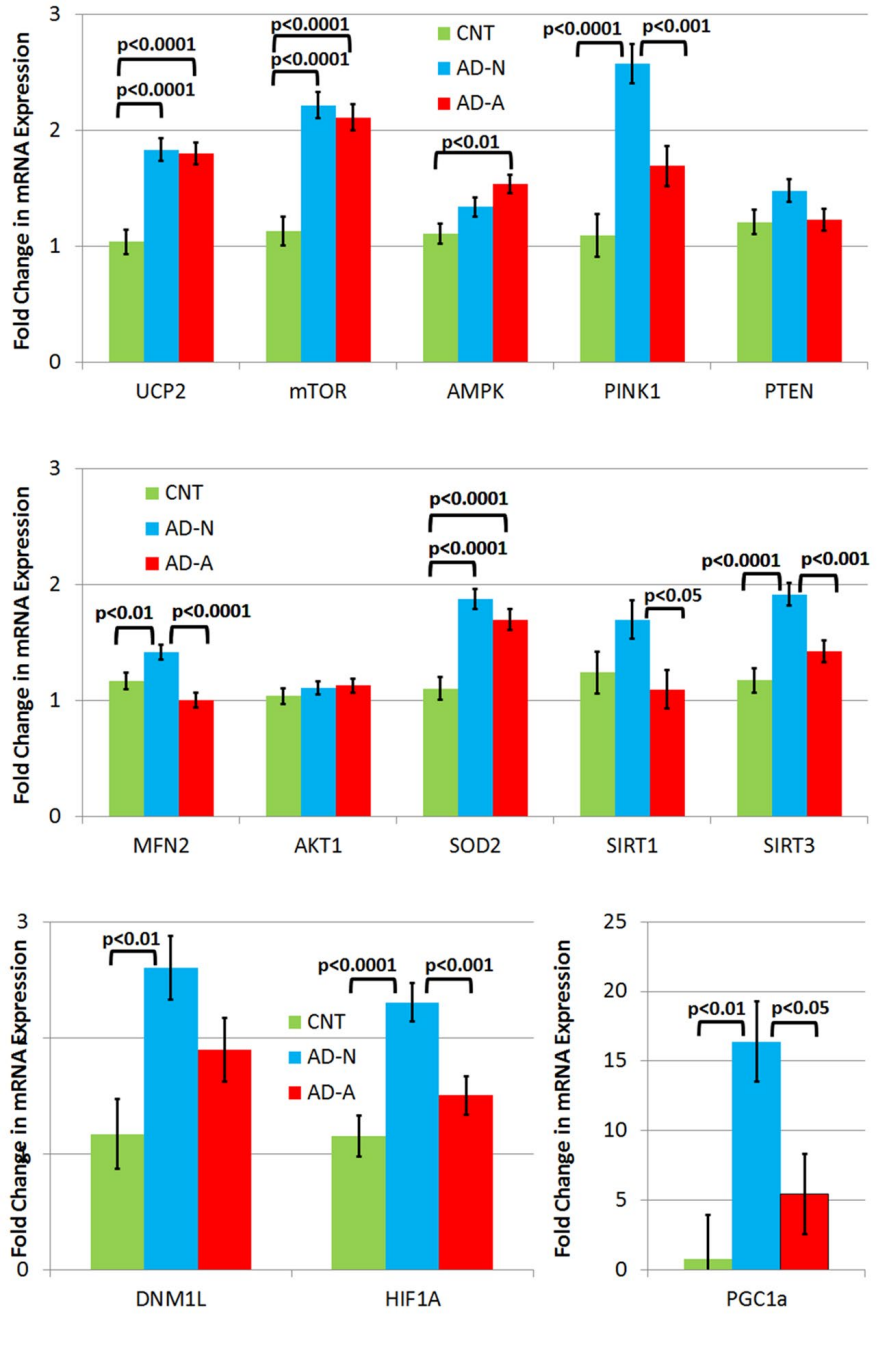
Gene expression in three different types of lymphobastoid cell lines (LCLs) collapsed across the prolonged (96 h) ROS exposure condition. While LCLs derived from individuals with autistic disorder (AD) demonstrated increases in expression in several genes, including UCP2, mTOR, and SOD2, in comparison to controls (CNT), most of the elevation in gene expression related to the genes involved in response to physiological stress was seen only one AD LCL group (i.e., AD-N).

To understand differences in molecular pathway regulation of mitochondrial and redox homeostasis across LCL groups, expression of key genes was examined (**Figure 3**). Gene expression was averaged across ROS exposure since differences in gene expression between AD and CNT LCL groups were independent of the ROS effect except for one gene.

#### No Differences

There were no differences in PTEN and AKT1 expression between LCL groups.

#### Gene Expression Elevated in Autistic Disorder Lymphoblastoid Cell Lines Compared to Control Lymphoblastoid Cell Lines

UCP2 expression was significantly different across LCL groups [*F*(2,44) = 19.12, *p* < 0.0001] due to higher expression in AD-N [*t*(44) = 5.54, *p* < 0.0001] and AD-A [*t*(44) = 5.31, *p* < 0.0001] LCLs as compared to CNT LCLs. SOD2 expression was significantly different across LCL groups [*F*(2,44) = 19.78, *p* < 0.0001] due to higher expression in AD-N [*t*(44) = 6.07, *p* < 0.0001] and AD-A [*t*(44) = 4.68, *p* < 0.0001] LCLs as compared to CNT LCLs. mTOR expression was significantly different across LCL groups [*F*(2,44) = 26.04, *p* < 0.0001] due to higher expression in AD-N [*t*(44) = 6.63, *p* < 0.0001] and AD-A [*t*(44) = 5.99, *p* < 0.0001] LCLs as compared to CNT LCLs.

#### Gene Expression Elevated in AD-A Lymphoblastoid Cell Lines

AMPK expression was significantly different across LCL groups [*F*(2,44) = 6.53, *p* < 0.005] due to higher expression in the AD-A LCL as compared to CNT LCLs [*t*(44) = 3.61, *p* < 0.005].

#### Gene Expression Elevated in AD-N Lymphoblastoid Cell Lines

PINK1 expression was significantly different across LCL groups [*F*(2,44) = 19.78, *p* < 0.0001] due to higher expression in AD-N LCLs as compared to CNT [*t*(44) = 6.20, *p* < 0.0001] and AD-A [*t*(44) = 3.89, *p* < 0.0005] LCLs. HIF1α expression was significantly different across LCL groups [*F*(2,44) = 11.99, *p* < 0.0001] due to higher expression in AD-N LCLs as compared to CNT [*t*(44) = 4.71, *p* < 0.0001] and AD-A [*t*(44) = 3.42, *p* = 0.001] LCLs. SIRT3 expression was significantly different across LCL groups [*F*(2,44) = 14.08, *p* < 0.0001] due to higher expression in AD-N LCLs as compared to CNT [*t*(44) = 5.16, *p* < 0.0001] and AD-A [*t*(44) = 3.56, *p* < 0.001] LCLs. PCG1α expression was significantly different across LCL groups [*F*(2,44) = 7.42, *p* = 0.01] due to higher expression in AD-N LCLs as compared to CNT [*t*(44) = 3.70, *p* < 0.005] and AD-A [*t*(44) = 2.73, *p* < 0.05] LCLs. MFN2 expression was significantly different across LCL groups [*F*(2,44) = 10.66, *p* < 0.0005] due to higher expression in AD-N LCLs as compared to CNT [*t*(44) = 2.60, *p* = 0.01] and AD-A [*t*(44) = 4.60, *p* < 0.0001] LCLs. SIRT1 expression was significantly different across LCL groups [*F*(2,44) = 3.58, *p* < 0.05] due to higher expression in AD-N LCLs as compared to AD-A [*t*(44) = 2.58, *p* < 0.05] LCLs. DNM1L was significantly different across LCL groups [*F*(2,44) = 6.18, *p* < 0.005] due to higher expression in AD-N LCLs as compared to CNT [*t*(44) = 3.51, *p* < 0.005] LCLs.

#### Gene Expression Changes Resulting From Prolonged Reactive Oxygen Species Exposure

There was also an LCL group by prolonged ROS exposure interaction [*F*(2,44) = 3.24, *p* = 0.05] driven by the fact that MFN2 (fusion) expression decreased in the AD-N LCLs while it increased in the CNT [*t*(44) = 2.27, *p* < 0.05] and AD-A [*t*(44) = 2.10, *p* < 0.05] LCLs following prolonged ROS exposure.

### Gene Expression Patterns Differentiate Lymphoblastoid Cell Line Groups: Visual Analysis

**Figure 4:**
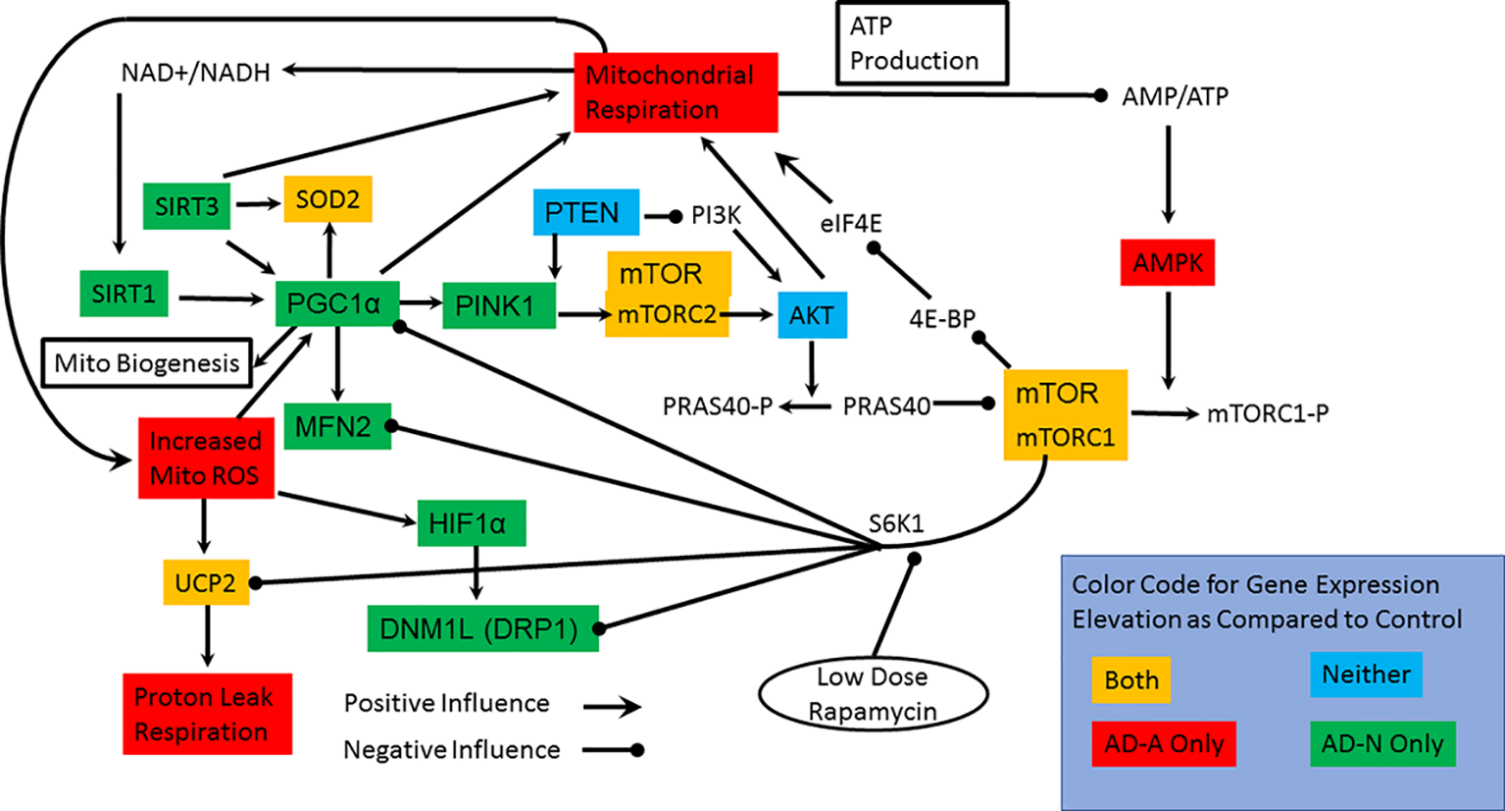
Interconnection of the pathways studied, the genes in which we have measured expression and connection to mitochondrial respiration. The colors represent the groups that have elevations in the expression of specific genes. Most notable are the two pathways by which mTOR can influence mitochondrial function. S6K1 pathway inhibits many of the pathways involved in mitochondrial maintainance and response to stress while the 4E-BP/eIF4E pathway more directly can increase mitochondrial function.

To better understand gene expression that differentiates LCL groups, particularly LCLs with and without mitochondrial dysfunction, the statistical differences found above were displayed in a functional diagram representing pathway interconnections (**Figure 4**). This diagram shows that ASD LCLs, both AD-A and AD-N LCLs, demonstrate an increase in SOD and UCP2, as would be expected for LCLs under chronic oxidative stress, as has been shown for ASD LCLs previously in several studies (37, 41, 61). One pattern that differentiates AD-A from AD-N is that many genes associated with mitochondrial maintenance (DRP1, MFN2) and response to physiological stress (PCG1α) are only elevated for AD-N LCLs but not AD-A LCLs. This is surprising, as the mitochondria with more abnormal respiratory rates would be expected to be under greater physiological stress.

For both types of ASD LCLs, mTOR is increased, which could represent either the mTORC1 or mTORC2 complex, but here we concentrate on mTORC1 because of its interactions with other pathways. The S6K1 pathway can be promoted by mTORC1. Since S6K1 inhibits many of the pathways involved in mitochondrial maintenance and response to stress, an increase in S6K1 pathway in the AD-A LCLs could explain why expression of many of the genes responsible for mitochondrial maintenance and response to stress are not elevated. The effect of S6K1 specific to mitochondrial dysfunction in the AD-A LCLs will be tested in the rapamycin experiment below. Why activation of the S6K1 pathway would be greater in the AD-A LCLs than in the AD-N LCLs is not immediately obvious, but this may be due to the differences in the dynamics of mTOR in the two ASD LCL subsets. Indeed, AMPK elevation appears to be specific for AD-A LCLs and would result in a greater phosphorylation and inactivation of mTORC1. Higher AMPK levels could explain the increase in mTOR expression for the AD-A LCLs since greater inactivation of mTORC1 could drive greater production of unphosphorylated mTORC1. The elevation in mTOR expression in AD-N LCLs could be driven by PINK1, but we would expect AKT1 to also be increased in expression.

### Gene Expression Patterns Differentiate Lymphoblastoid Cell Line Groups: Discriminant Analysis

To complement the qualitative description of the different patterns that differentiate the LCL groups, a machine learning technique was used to independently verify patterns unique to the LCL groups. Since the expression between many genes are interdependent, PLSDA was used to select the key genes that differentiate LCL characteristics, specifically whether the LCLs came from individuals with ASD or those with typical development, and whether or not the mitochondria in the LCLs manifested mitochondrial dysfunction. Thus, we used PLSDA to extract the most important genes that defined these specific characteristics. PLSDA was used as compared to other discriminant analysis procedures since it considers the relationship between genes as well as their absolute expression levels.

#### Autism vs. Nonautism

Overall accuracy of the canonical discriminant function was 97% using two components that accounted for 56% and 13% of the variance. The most significant component loadings were represented by six genes: mTOR, UCP2, PTEN, AKT1, SIRT1, and MFN2.

#### Mitochondrial Dysfunction vs. Normal Mitochondrial Function

Overall accuracy of the canonical discriminant function was 84% using two components that accounted for 51% and 11% of the variance. The most significant component loadings were represented by six genes: mTOR, UCP2, DRP1, AMPK, SIRT1, and MFN2.

Thus, the PLSDA demonstrated that several common genes differentiated the bioenergetic and clinical characteristics of the cells, specifically mTOR, UCP2, SIRT1, and MFN2. Two genes were more specific to the clinical characteristics of the patients from which the LCLs were derived, specifically PTEN and AKT1, and two genes where more specific for the bioenergetic characteristics of the LCLs, specifically AMPK and DRP1.

### Changes in Mitochondrial Respiration With Rapamycin Treatment

**Figure 5:**
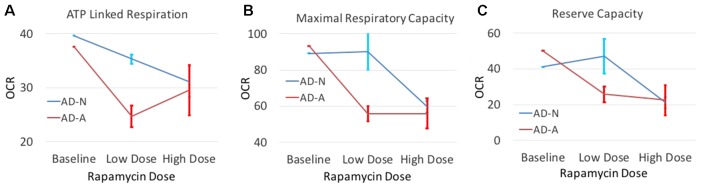
The effect of both low-dose and high-dose rapamycin on one pair of ASD LCLs. LCLs were exposed to low dose (0.01 nM and 0.1 nM) or high dose (1 nM and 10 nM) for either 1 h or 2 h followed by the Seahorse assay. For presentation purposes, both low doses at both time points were combined into one average data point and both high doses at both time points were combined into one data point. Standard error bars are also shown. Respiratory parameters including **(A)** ATP Linked Respiration, **(B)** Maximal Respiratory Capacity and **(C)** Reserve Capacity are shown.

In order to determine if modulation of S6K1 could affect mitochondrial function, we investigated whether low-dose rapamycin did indeed differentiate the two ASD LCL groups. First, we did a titration experiment to find the optimal low dose of rapamycin and to confirm that low-dose but not high-dose rapamycin differentially influenced the two ASD groups. As seen in **Figure 5**, low-dose rapamycin markedly reduced the respiratory indexes in the AD-A LCLs but not the AD-N LCLs as expected. From these experiments, we selected the optimal low dose to examine this effect on a larger number of LCL pairs.

Mitochondrial function was measured in eight pairs of LCLs from the two AD LCL groups (AD-N and AD-A) with and without exposure to low-dose (0.1 nM) rapamycin since this concentration of rapamycin has a more selective effect on the regulation of S6K1 by mTOR (59). We hypothesized that the activation of S6K1 by mTORC1 suppresses genes for mitochondrial maintenance and response to stress and that by decreasing activity of this pathway using low-dose rapamycin, mitochondrial dysfunction in the AD-A LCLs will normalize.

**Figure 6:**
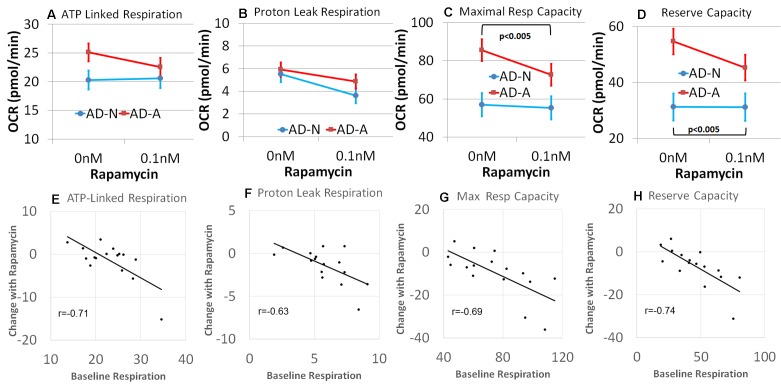
Rapamycin modulates mitochondrial function differently depending on the underlying baseline repiratory rate in lymphobastoid cell lines (LCLs) derived from children with autistic disorder (AD). **(A–D)** Group differences in the effect of rapamycin. The AD-N LCLs demonstrate more typical mitochondrial function and have less influence from rapamycin while the AD-A LCLs, which demonstrate elevated mitochondrial function, are more influenced by rapamycin. **(E–H)** The linear relationship between the underlying baseline respiratory rate and the change in respirataory rate for both groups of LCLs. The higher the baseline respiratory rate, the greater the effect of rapamycin.

Analyzing the results by LCL group, rapamycin was found to decrease PLR [*F*(1,13) = 7.37, *p* < 0.05] but not ALR (**Figure 6A** and **B**). Consistent with our hypothesis, rapamycin significantly decreased MRC [*F*(1,13) = 20.06, *p* < 0.001] and RC [*F*(1,13) = 23.41, *p* < 0.001], with this effect significantly greater for the AD-A LCLs [MRC: *F*(1,13) = 12.07, *p* < 0.005; RC: *F*(1,13) = 23.05, *p* < 0.001] as compared to the AD-N LCLs (**Figure 6C** and **D**). In fact, for AD-A LCLs, MRC and RC decreased toward the AD-N respiratory parameters indicating normalization to these respiratory parameters.

We then determined if the effect of low-dose rapamycin was dependent on baseline mitochondrial respiration independent of the LCL grouping, by examining the relationship between the baseline respiratory parameters and the change in the respiratory parameter with rapamycin exposure. A significant relationship between the baseline respiratory parameter and change in the respiratory parameter with rapamycin treatment was found for all respiratory parameters [ALR: *F*(1,12) = 7.85, *p* < 0.05, *r* = −0.71; PLR: *F*(1,12) = 14.35, *p* < 0.005, *r* = −0.63; MRC: *F*(1,12) = 8.20, *p* = 0.01, *r* = −0.69; RC: *F*(1,12) = 12.77, *p* < 0.005, *r* = −0.74; **Figure 6E–H**]. In general, rapamycin had a greater effect on reducing mitochondrial respiration for those LCLs with higher respiratory rates at baseline. All correlation coefficients were significant, and these relationships were not significantly different across AD groups. Thus, in general, it appeared that rapamycin had a greater effect on reducing mitochondrial respiration when mitochondrial respiratory rates were higher.

## Discussion

This study examines important aspects of mitochondrial function and the origins of atypical mitochondrial respiration previously observed in a subset of ASD LCL in at least seven independent studies (36–42). In our previous studies, we hypothesized that ASD LCLs with atypical mitochondrial respiration, called AD-A LCLs, developed atypical mitochondrial respiration as an adaptation to previous environmental exposures, potentially through prolonged exposure to ROS since oxidative stress is a common mechanism in which environmental agents perturb cellular physiology.

In this study, we conducted three experiments: 1) we examined the effect of prolonged exposure to ROS on mitochondrial respiration; 2) we examined differences in the expression of genes important for mitochondrial respiration, particularly those involved in allowing the mitochondria to adapt to adverse physiological conditions, across different clinical types (ASD vs. typical developing) and physiological types (atypical vs. normal mitochondrial function); and 3) we also examined the effect of low-dose rapamycin on mitochondrial function, to determine whether a specific regulatory pathway (S6K1) may be involved in maintaining atypical mitochondrial function in the AD-A LCLs.

First, we examined the effect of prolonged exposure to low concentrations of an agent (DMNQ) that increased intracellular ROS to alter baseline mitochondrial respiration. We examined this in three types of LCLs, those derived from typically developing children (CNT) and two types from children with ASD. One of the subsets of LCLs derived from children with ASD are known to have atypical mitochondrial respiration (AD-A), which has been hypothesized to be an adaptive response to previous exposures to environmental stressors involving ROS. The prolonged exposure to ROS did result in changes in mitochondrial respiration, some of which were more significant and marked in the AD LCLs, but, overall, did influence the CNT LCLs also. These changes were similar to the differences seen in the AD-A LCLs at baseline, suggesting that the pattern of mitochondrial dysfunction displayed in the AD-A LCLs may have arisen from previous prolonged exposure to ROS.

Interestingly, the analysis of changes in gene expression with prolonged exposure to ROS found that MFN2 (fusion) gene expression changed, although in different directions for the different LCLs groups. While MFN2 expression decreased for AD-N LCLs and increased for both AD-A and CNT LCLs, it needs to be noted that the AD-N LCLs showed a higher expression of this gene at baseline. What is also potentially significant is that MFN2 appears to be significant in differentiating both clinical groups and bioenergetic characteristics of the LCLs, pointing to the potential importance of the maintenance of optimal mitochondria function in ASD. It is also of interest that the AD-A LCLs demonstrated significantly lower MFN2 at baseline as compared to the AD-N LCLs. Thus, in lowering MFN2 expression for AD-N LCLs, prolonged ROS exposure did alter MFN2 toward the baseline expression of the AD-A LCLs, suggesting that this difference in MFN2 expression may be an integral part of the regulation and maintenance of the atypical mitochondrial activity.

Second, we examined gene expression in three types of LCLs that have different intrinsic mitochondrial function and underlying intracellular ROS profiles. Both types of ASD LCLs demonstrated increased expression of UCP2, SOD2, and mTOR. As both types of ASD LCLs are known to have increased intracellular ROS, it is not surprising that UCP2 and SOD2 were increased, although our previous study suggested that UCP2 was higher in AD-A as compared to AD-N LCL when the protein content was measured (37). Further, since glutathionylation (62) activates UCP2, relative changes in glutathione in the ASD LCLs also likely modulate UCP2 function. Thus, UCP2 gene expression between ASD LCLs only provides one aspect of its function in LCLs under chronic ROS.

The AD-N LCLs clearly demonstrated an increase in several genes involved in mitochondrial response to stress and mitochondrial dynamics aimed at improving mitochondrial fidelity. Previous studies have demonstrated that overexpression of PCG1α in ASD LCLs results in upregulation of mitochondrial ETC Complex I and III as well as reduces mitochondrial ROS (54). Thus, AD-N LCLs appear to have pathways for supporting mitochondrial response to stress even without an exogenous ROS challenge. The fact that the AD-A LCLs did not demonstrate an increase in these genes was surprising and may suggest that their abnormal mitochondrial function is an alternative adaptation to the chronic intracellular ROS. Visual analysis of pathways suggested that the S6K1 pathway, which is activated by mTORC1, could suppress these pathways and account for a suppression of pathways important for mitochondrial response to stress and maintenance. Our third experiment tests this hypothesis.

The AD-A LCLs also demonstrated an increase in AMPK expression, which is unexpected as AMPK is inhibited by elevation in intracellular ATP, and from our previous studies, the AD-A appears to be overproducing ATP. This result would appear to suggest that despite increased production of ATP, AD-A LCLs remain at an overall ATP deficit or, alternatively, an upstream signal is activating AMPK. Either way, this elevation in AMPK can significantly affect mTORC1 levels in the LCLs as higher AMPK will enhance the phosphorylation of mTORC1, thereby inactivating its influences on downstream signals. This could explain the increase in mTOR expression for the AD-A LCLs as more mTOR would need to be produced to maintain an active mTORC1 complex.

Third, we examined the effect of low-dose rapamycin on parameters of mitochondrial respiration. We found that overall rapamycin decreased MRC and RC, with this decrease much greater in the LCLs with higher respiration, the AD-A LCLs, as compared to those with lower respiratory rates, the AD-N LCLs. We also found that the effect of low-dose rapamycin in decreasing respiratory parameters was proportional to the amount they were increased at baseline, so that the higher the baseline respiratory parameter value, the more low-dose rapamycin decreased the parameter. This appears to be consistent across the four respiratory parameters measured. Others have found that low-dose rapamycin modulates the effect of mTOR on S6K1. S6K1 inhibits several of the genes that are elevated in the AD-N but not the AD-A LCLs. Thus, by decreasing the positive influence of mTOR on S6K1, the inhibitory effect on genes associated with mitochondrial response to stress and mitochondria maintenance could be uninhibited. If these mitochondrial regulatory pathways were responsible for maintaining normal mitochondrial respiration in the context of chronic oxidative stress, uninhibiting them could normalize mitochondrial function in the AD-A LCLs. This is exactly what was found. However, the effect of normalizing mitochondrial respiration was proportional to the abnormal elevation in mitochondrial respiration regardless of whether the LCLs were in the AD-A or AD-N group. Thus, this does suggest that the abnormalities in mitochondrial respiration may be on a continuum. Such a notion would be consistent with the fact that chronic exposure to ROS can change the respiratory characteristics of LCLs toward this atypical pattern with the magnitude of this change different for the different LCL groups.

mTORC1 is known to stimulate mitochondrial ATP production (57, 63), but both ASD LCLs demonstrate similar mTOR gene expression. The AD-A LCLs demonstrate elevated expression of AMPK that phosphorylates regulatory-associated protein of mTOR (RAPTOR), inhibiting its activity. This would be expected to decrease the activity of the mTOR pathway. However, the rapamycin experiments suggest that the mTOR pathway is relevant to mitochondrial function in AD-A LCLs. It is possible that the increased sensitivity to rapamycin and the preferential activation of the S6K1 pathways as compared to the 4E-BP pathway by mTORC1 in AD-A LCLs are due to the fact that it has a more narrow range of activation because it is chronically inhibited by a chronic increase in AMPK.

### The Role of Mitochondrial Dysfunction in Neurodevelopmental Disorders

Interestingly, the pattern of a significant elevation in mitochondrial respiratory activity found in the AD-A LCLs is not unprecedented and has been associated with genetic syndromes and models of ASD resulting from environmental influences. For example, genetic syndromes associated with ASD, including patients with Phelan–McDermid syndrome (33), WDR45 (17), 22q13 duplication (21), and Rett syndrome (18), as well as the PTEN haploinsufficient mouse model of ASD (16) have demonstrated significant elevations in ETC function. Also, brain tissue from the maternal immune activation (MIA) mouse, a model of ASD induced by prenatal environmental stress, has overactivity in Complex I and IV (64). This abnormal pattern of mitochondrial function has also been associated with other disorders. For example, ETC Complex IV underactivity is found in chronic multiple sclerosis lesions during inflammation but ETC Complex IV has been found to be overactive in these lesions once inflammation has resolved, suggesting that inflammation had a long-term effect on mitochondrial activity (65, 66). Significantly, this “hyperactive” state of ETC Complex IV has been described during reperfusion following ischemia and is believed to contribute to the increased production of ROS during reperfusion (67). Thus, a better understanding of this atypical increase in respiratory rate and its role in disease may have significant consequences.

### The Link Between Environmental Factors and Mitochondrial Function

Interestingly, consistent with the chronic ROS exposure experiment, we have found that environmental influences associated with ASD can alter mitochondrial function in CNT LCLs to make them more like AD-A LCLs, thereby supporting our original hypothesis. For example, in a recent study, we demonstrated that prolonged exposure to trichloroacetaldehyde hydrate, the active metabolite of an environmental toxicant associated with ASD, can alter CNT LCLs to have mitochondrial function like AD-A LCLs and that AD-A LCLs are more resilient to the detrimental effect of trichloroacetaldehyde hydrate on the mitochondria as compared to AD-N LCLs (40). In addition, further studies have implicated the enteric microbiome-derived short-chain fatty acids butyrate (42) and propionic acid (39) in increasing respiratory rates of mitochondria in LCLs from children with ASD.

Overall, we believe that the experiments reported in this manuscript may provide insight into adaptive changes in mitochondrial function that are only starting to be recognized. We believe that these changes may be caused by environmental influences in many cases and the information uncovered may be helpful in understanding environmentally induced diseases in greater depth. In addition, it appears that the mTOR pathway may have a role in modulating changes in mitochondrial function in the context of these atypical changes in mitochondrial function and may have a role in developing novel treatments.

## Author Contributions

All authors were involved in the design and conceptualization of the experiments. Laboratory experiments were conducted by SB and SR. Data were analyzed by all authors. All authors were involved in drafting, editing, and finalizing the manuscript. All authors approved the final manuscript.

## Conflict of Interest Statement

The authors declare that the research was conducted in the absence of any commercial or financial relationships that could be construed as a potential conflict of interest.
