# Supplementary material for: Mitochondrial Dysfunction Is Inducible in Lymphoblastoid Cell Lines From Children With Autism and May Involve the TORC1 Pathway
Source: Front Psychiatry. 2019 May 7;10:269. doi: 10.3389/fpsyt.2019.00269 (PMC6514096; doi:10.3389/fpsyt.2019.00269)
Supplement: Supplementary file 1 [file DataSheet_1.docx]

**Supplementary** **Tables**

| **Supplementary Table 1.** Lymphoblastoid Cell Lines used in this study. Note three types of cell lines were used with two types of Autistic Disorder cell lines which were characterized in our previous studies. | | | | | | | | |
| --- | --- | --- | --- | --- | --- | --- | --- | --- |
| **Controls** | | | **AD-N Subgroup** | | | **AD-A Subgroup** | | |
| **Cell ID** | **Source** | **Age (y)** | **Cell ID** | **Source** | **Age (y)** | **Cell ID** | **Source** | **Age (y)** |
| GM09622 | Coriell | 7 | 02C10618 | NIMH | 7 | 03C14441 | NIMH | 7 |
| GM09659 | Coriell | 4 | 04C24363 | NIMH | 4 | AU1393306 | AGRE | 3 |
| GM10153 | Coriell | 10 | 03C17237 | NIMH | 10 | AU0939303 | AGRE | 11 |
| GM18054 | Coriell | 5 | 03C15992 | NIMH | 5 | 01C08495 | NIMH | 4 |
| GM16007 | Coriell | 12 | 05C38988 | NIMH | 12 | AU1165302 | AGRE | 13 |
| GM09380 | Coriell | 6 | 01C08022 | NIMH | 5 | 01C08495 | NIMH | 4 |
| GM09642 | Coriell | 7 | 04C27439, | NIMH | 7 | 02C09713 | NIMH | 7 |
| GM09642 | Coriell | 7 | 01C08367 | NIMH | 7 | 01C08594 | NIMH | 7 |
| GM11626 | Coriell | 13 | AU008404 | AGRE | 13 | AU1165302 | AGRE | 13 |
| GM10153 | Coriell | 10 | 00C04757 | NIMH | 10 | AU0939303 | AGRE | 11 |
| GM11973 | Coriell | 7 | 02C09650 | NIMH | 7 | 02C09713 | NIMH | 7 |
| GM11599 | Coriell | 9 | AU038804 | AGRE | 8 | 03C14441 | NIMH | 7 |
| GM10153 | Coriell | 10 | AU1267302 | AGRE | 10 | 03C16499 | NIMH | 11 |

Coriell = Coriell Cell Repository (Camden, NJ, USA); NIMH = National Institutes of Mental Health Biorepository (Bethesda, MD, USA); AGRE= Autism Genetic Resource Exchange Biorepository (Los Angeles, CA, USA)

| **Supplementary Table 2.** Lymphoblastoid Cell Lines used for the rapamycin portion of the study. | | | | | |
| --- | --- | --- | --- | --- | --- |
| **AD-N Subgroup** | | | **AD-A Subgroup** | | |
| **Cell ID** | **Source** | **Age (y)** | **Cell ID** | **Source** | **Age (y)** |
| 02C10618 | NIMH | 7 | 03C14441 | NIMH | 7 |
| 02C10054 | NIMH | 6 | 01C08594 | NIMH | 7 |
| 04C26296 | NIMH | 10 | 03C16499 | NIMH | 11 |
| 03C14363 | NIMH | 4 | AU1393306 | AGRE | 3 |
| 00C04757 | NIMH | 10 | AU0939303 | AGRE | 11 |
| AU008404 | AGRE | 13 | AU1165302 | AGRE | 13 |
| 02C09650 | NIMH | 7 | 02C09713 | NIMH | 7 |
| 01C08022 | NIMH | 5 | 01C08495 | NIMH | 4 |

| **Supplementary Table 3.** Lymphoblastoid Cell Lines used for gene expression portion of the study. Note three types of cell lines were used with two types of Autistic Disorder cell lines which were characterized in our previous studies. | | | | | | | | |
| --- | --- | --- | --- | --- | --- | --- | --- | --- |
| **Controls** | | | **AD-N Subgroup** | | | **AD-A Subgroup** | | |
| **Cell ID** | **Source** | **Age (y)** | **Cell ID** | **Source** | **Age (y)** | **Cell ID** | **Source** | **Age (y)** |
| GM09622 | Coriell | 7 | 02C10618 | NIMH | 7 | 03C14441 | NIMH | 7 |
| GM17255 | Coriell | 6 | 02C10054 | NIMH | 6 | 01C08594 | NIMH | 7 |
| GM09380 | Coriell | 6 | 01C08022 | NIMH | 5 | 01C08495 | NIMH | 4 |
| GM09659 | Coriell | 4 | 04C24363 | NIMH | 4 | AU1393306 | AGRE | 3 |
| GM10153 | Coriell | 10 | 00C04757 | NIMH | 10 | AU0939303 | AGRE | 11 |
| GM09642 | Coriell | 7 | 01C08367 | NIMH | 7 | 01C08594 | NIMH | 7 |
| GM11973 | Coriell | 7 | 02C09650 | NIMH | 7 | 02C09713 | NIMH | 7 |
| GM09642 | Coriell | 7 | 04C27439 | NIMH | 7 | 02C09713 | NIMH | 7 |
| GM16007 | Coriell | 12 | 05C38988 | NIMH | 12 | AU1165302 | AGRE | 13 |
| GM16007 | Coriell | 12 | AU1215301 | AGRE | 12 | 03C16499 | NIMH | 11 |
| GM17272 | Coriell | 17 | 03C14349 | NIMH | 17 | AU1165302 | AGRE | 13 |
| GM10153 | Coriell | 10 | AU1267302 | AGRE | 10 | 03C16499 | NIMH | 11 |
| GM10153 | Coriell | 10 | 03C17237 | NIMH | 10 | AU0939303 | AGRE | 11 |

Coriell = Coriell Cell Repository (Camden, NJ, USA); NIMH = National Institutes of Mental Health Biorepository (Bethesda, MD, USA); AGRE= Autism Genetic Resource Exchange Biorepository (Los Angeles, CA, USA)

| Supplementary Table 4. List of primer sequences used for real time polymerase chain reaction analysis. F: Forward. R: Reverse. | | |
| --- | --- | --- |
| Genes | NLM ID | Primer sequences |
| HPRT1 | NM_000194 | F: 5' - TGCTGAGGATTTGGAAAGGG - 3' |
|  |  | R: 5' - ACAGAGGGCTACAATGTGATG - 3' |
| UCP2 | NM_003355 | F: 5' - TCCTGAAAGCCAACCTCATG - 3' |
|  |  | R: 5' - GGCAGAGTTCATGTATCTCGTC - 3' |
| MTOR | NM_004958 | F: 5' - CAAGAACTCGCTGATCCAAATG - 3' |
|  |  | R: 5' - GCTGTACGTTCCTTCTCCTTC - 3' |
| PINK1 | NM_032409 | F: 5' - GAGTATGGAGCAGTCACTTACAG - 3' |
|  |  | R: 5' - CAGCACATCAGGGTAGTCG - 3' |
| MFN2 | NM_014874 | F: 5' - ATGTGGCCCAACTCTAAGTG - 3' |
|  |  | R:5' - CACAAACACATCAGCATCCAG - 3' |
| CREB1 | NM_004379 | F: 5' - TTCTACAGTATGCACAGACCAC - 3' |
|  |  | R: 5' - ATGCCATAACAACTCCAGGG - 3' |
| HIF1A | NM_001530 | F: 5' - AACATAAAGTCTGCAACATGGAAG - 3' |
|  |  | R: 5' - TTTGATGGGTGAGGAATGGG - 3' |
| PGC1 (PPARGC1A) | NM_013261 | F: 5' - CAGGCAGTAGATCCTCTTCAAG - 3' |
|  |  | R: 5' - TCCTCGTAGCTGTCATACCTG - 3' |
| DNM1L (DRP1) | NM_005690 | F: 5' - TTCCATTATCCTCGCTGTCAC - 3' |
|  |  | R: 5' - CATCAGTACCCGCATCCATG - 3' |
| AMPK | NM_006251 | F: 5' - CTCAGTTCCTGGAGAAAGATGG - 3' |
|  |  | R: 5' - CCCAGTCAATTCATGTTTGCC - 3' |
| PTEN | NM_000314 | F: 5' - AAGGGACGAACTGGTGTAATG - 3' |
|  |  | R: 5' - GCCTCTGACTGGGAATAGTTAC - 3' |
| AKT1 | NM_001014431 | F: 5' - 5TCTATGGCGCTGAGATTGTG - 3' |
|  |  | R: 5' - TCTTAATGTGCCCGTCCTTG - 3' |
| SOD2 | NM_000636 | F: 5' - CCTGGAACCTCACATCAACG - 3' |
|  |  | R: 5' - GCTATCTGGGCTGTAACATCTC - 3' |
| SIRT1 | NM_012238 | F: 5' - CCCTCAAAGTAAGACCAGTAGC - 3' |
|  |  | R: 5' - CACAGTCTCCAAGAAGCTCTAC - 3' |
| SIRT3 | NM_012239 | F: 5' - TCATGGAACCTTTGCCTCTG - 3' |
|  |  | R: 5' - GCTCCCCAAAGAACACAATG - 3' |
